# A phase II study of TAS-117 in patients with advanced solid tumors harboring germline *PTEN*-inactivating mutations

**DOI:** 10.2217/fon-2022-0305

**Published:** 2022-08-26

**Authors:** Jordi Rodón, Pauline Funchain, Theodore W Laetsch, Hendrik-Tobias Arkenau, Alice Hervieu, Christian F Singer, Yonina R Murciano-Goroff, Sant P Chawla, Kristin Anthony, Ikuo Yamamiya, Mei Liu, Abdel-Baset Halim, Karim A Benhadji, Osamu Takahashi, Suzette Delaloge

**Affiliations:** 1 Department of Investigational Cancer Therapeutics, Division of Cancer Medicine, MD Anderson Cancer Center, Houston, TX 77030, USA; 2 Cleveland Clinic, Cleveland, OH 44195, USA; 3 Children's Hospital of Philadelphia, & University of Pennsylvania, Philadelphia, PA 19104, USA; 4 Sarah Cannon Research Institute, London, UK, & Cancer Institute, University College London, London, W1G 6AD, UK; 5 Centre Georges-François Leclerc, Dijon, France; 6 Dept of OB/GYN & Comprehensive Cancer Center, Medical University of Vienna, Vienna, 1090, Austria; 7 Memorial Sloan Kettering Cancer Center, New York City, NY 10065, USA; 8 Sarcoma Oncology Research Center, Santa Monica, CA 90403, USA; 9 The PTEN Hamartoma Tumor Syndrome Foundation, Huntsville, AL 35806, USA; 10 Taiho Oncology, Princeton, NJ 08540, USA; 11 Institut Gustave Roussy, Villejuif, 94805, France

**Keywords:** advanced solid tumor, clinical trial, Cowden syndrome, germline *PTEN* mutation, PTEN hamartoma tumor syndrome, PTEN inactivation, TAS-117

## Abstract

PTEN acts as a potent tumor suppressor within the PI3K/AKT/mTOR pathway. Germline mutations in the *PTEN* gene are a hallmark of PTEN hamartoma tumor syndrome, which includes Cowden syndrome, where they appear to elevate lifetime risk of cancer. Targeted AKT directed therapy has been proposed as an effective approach in cancer patients having germline *PTEN* mutations. The mechanism of action, safety and dosing regimen for the novel allosteric AKT inhibitor TAS-117 have been explored in a phase I study in Japan in which activity was observed against certain tumor types. Here we describe the study protocol of an international, two-part phase II study evaluating the safety, tolerability, pharmacokinetics, pharmacodynamics and antitumor activity of TAS-117 in patients with advanced solid tumors harboring germline *PTEN*-inactivating mutations.

## Plain language summary

Signaling paths control growth and activities inside cells. Overactivity in these paths can encourage many types of cancers to develop. Tumor suppressor proteins can inhibit cell signals that promote cancer. This protection can be lost if there are errors in any gene coding for a tumor suppressor protein. We are carrying out a clinical study to test TAS-117, a potential new oral medicine, in people who have solid tumors and whose cells have changes in their genes that inactivate a tumor suppressor protein called PTEN. TAS-117 targets part of a signaling path that may be overactive due to loss of PTEN activity. In early research, TAS-117 has shown promising activity against certain tumor types. Our trial will explore if TAS-117 can provide a new treatment for rare forms of cancer where genetic changes have led to a loss of PTEN activity.

**Clinical Trial Registration:** NCT04770246 (ClinicalTrials.gov)

**Figure 1.**
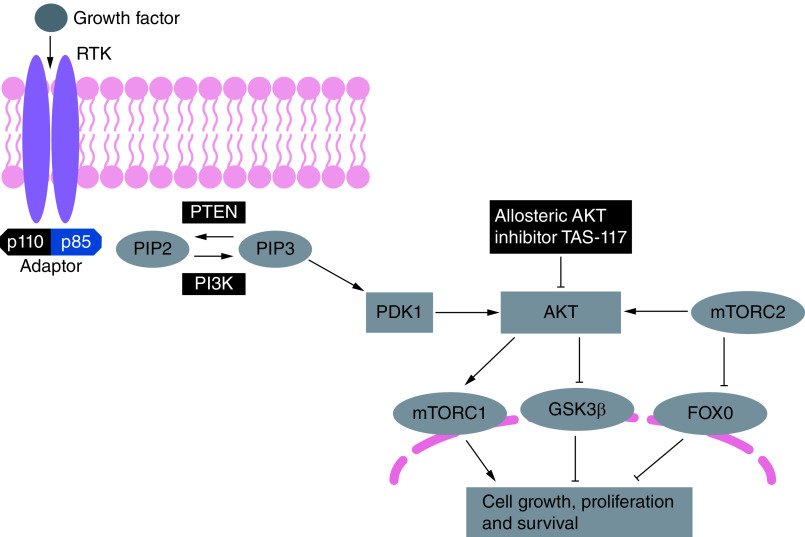
Mechanisms of AKT activation and signaling cascade. Stimulation of growth factor RTKs on the plasma membrane leads to activation of class IA PI3Ks. Activated class IA PI3Ks catalyze the conversion of PIP2 to the second messenger PIP3, in a reaction that can be reversed by PTEN. AKT and PDK1 bind PIP3 at the plasma membrane. AKT activation, in turn, activates mTORC1. Maximal AKT activation requires phosphorylation by mTORC2. AKT inhibits effector proteins via phosphorylation, including GSK3β and forkhead family of transcription factors (FOXO). The signaling results in the regulation of cell proliferation, survival and metabolism. Black arrows represent signaling activation while black bars indicate inhibitory signals. FOXO: Forkhead box subfamily O; GSK3β: Glycogen synthase kinase-3 beta; mTORC: mTOR complex; PDK1: Phosphoinositide-dependent kinase 1; PIP2: Phosphatidylinositol 4,5-bisphosphate; PIP3: Phosphatidylinositol 3,4,5-trisphosphate; PTEN: Phosphatase and tensin homolog deleted on chromosome 10; RTK: Receptor tyrosine kinase. Adapted with permission from [4].

Intracellular signaling through the PI3K/AKT/mTOR pathway plays a key role in regulating cell growth, motility, survival, metabolism and angiogenesis [1]. In an analysis of 19,784 tumor samples from 60 countries, aberrations in the PI3K/AKT/mTOR pathway were identified in 38% of solid tumors overall, with the highest frequency seen in endometrial cancer [2]. As aberrant activation of PI3K/AKT/mTOR signaling contributes to oncogenesis in solid tumors and hematologic malignancies, there is interest in developing therapies targeting components of this pathway [1,3]. One such target is AKT itself, a serine/threonine protein kinase, which is activated and exerts its effects through signaling pathways involving PI3K lipid kinases and the mTOR complexes 1 and 2 (mTORC1 and mTORC2) (Figure 1) [4].

Within the PI3K/AKT/mTOR pathway, alterations of phosphatase and tensin homolog deleted on chromosome 10 (PTEN) occur frequently in tumors (in one large study, 30% of 19,784 solid tumors analyzed were found to have PTEN protein loss by immunohistochemistry and 6% had mutations in the *PTEN* gene) [2]. PTEN acts as a potent tumor suppressor by attenuating PI3K signaling [5]. Loss of PTEN function is frequently observed in both heritable and sporadic cancers, and even a subtle decrease in PTEN levels and activity results in cancer susceptibility and favors tumor progression [6]. Germline mutations in *PTEN* have been reported in a variety of rare syndromes that are collectively known as PTEN hamartoma tumor syndrome (PHTS). PHTS features various benign and malignant tumors, as well as neurodevelopmental disorders, such as autism spectrum disorder [7]. Patients with germline PTEN mutations are often offered genetic counselling. Cowden syndrome (CS) is the best-described PHTS, with other forms, such as Bannayan–Riley–Ruvalcaba syndrome, Proteus syndrome (PS), Proteus-like syndrome and *PTEN* related autism spectrum disorder, also being associated with germline mutations in *PTEN* [7].

In CS, germline *PTEN* mutations inherited in an autosomal dominant manner are found in approximately 80% of cases [8]. Although it is a rare disease (estimated to occur in one in 200,000 births [9], CS is associated with increased lifetime risk of several cancers, such as breast, thyroid, renal and endometrial cancer (estimated lifetime risks of 85.2, 35.2, 34.0 and 28.2%, respectively) [10]. In patients with CS, *PTEN* mutations appear to drive an earlier onset of cancer, including forms that can present in pediatric patients [10,11]. Diagnosis of CS normally occurs between 13 and 65 years of age (see Table 1 for diagnostic criteria) [12]. Early identification of affected individuals, which often precedes development of advanced cancer by several years, allows appropriate surveillance to be instituted, which is key to timely detection of lesions. Individuals who are at risk of inheriting CS should; therefore, undergo genetic testing early in life to determine whether they require surveillance for PHTS related cancers. The frequency and type of surveillance should be aligned with current clinical guidelines [13,14].

**Table 1.** Diagnostic criteria for Cowden syndrome^†^.

Major criteria	Minor criteria
• Breast cancer • Endometrial cancer (epithelial) • Thyroid cancer (follicular) • GI hamartomas • Lhermitte–Duclos disease (adult) • Macrocephaly (≥97 percentile: 58 cm for females, 60 cm for males) • Macular pigmentation of glans penis • Multiple mucocutaneous lesions (any of the following): ○ ≥3 multiple trichilemmomas ○ ≥3 acral keratoses ○ ≥3 mucocutaneous neuromas • ≥3 oral papillomas	Autism spectrum disorder • Colon cancer • ≥3 esophageal glycogenic acanthosis • ≥3 lipomas • Mental retardation (i.e., IQ ≤75) • Renal cell carcinoma • Testicular lipomatosis • Thyroid cancer (papillary or follicular variant of papillary) • Thyroid structural lesions (e.g., adenoma, multinodular goiter) • Vascular anomalies (including multiple intracranial developmental venous anomalies)

† Three or more major criteria, including macrocephaly, Lhermitte–Duclos disease or GI hamartomas; or two major and three or more minor criteria are needed for a diagnosis of Cowden syndrome.

GI: Gastrointestinal; IQ: Intelligence quotient.

Adapted with permission from [12].

Management of patients with CS focuses on patient-specific findings, such as the presence of mucocutaneous lesions, breast carcinoma or nonmedullary thyroid cancer [15,16]. There are currently no approved therapies to target the PI3K/AKT/mTOR pathway in patients with cancers having germline *PTEN* mutations and CS or other forms of PHTS. Targeted AKT therapy has been proposed as an effective approach in cancer patients having germline *PTEN* mutations based on observations of durable complete tumor responses in two patients with metastatic breast cancer harboring different germline *PTEN* mutations who were treated with the catalytic AKT inhibitor capivasertib (both patients’ tumors had a total lack/markedly diminished PTEN staining) [8]. As well, a patient with Proteus syndrome has recently been reported to be successfully treated during 5 years with miransertib, another AKT inhibitor [17]. Therefore, we hypothesized that patients with advanced or metastatic cancer harboring germline *PTEN*-inactivating mutations may benefit from monotherapy with TAS-117, a novel allosteric AKT inhibitor.

The study by Millis *et al.* [2] found that 30% of 19,784 solid tumors analyzed were found to have PTEN protein loss by immunohistochemistry and 6% had mutations in the PTEN gene. The PTEN protein has multiple functions and highly complex modes of regulation [5]. This means that determining the underlying biologic basis of why a tumor is not expressing PTEN is not straightforward unless the tumor is found to harbor a PTEN inactivating mutation. Combined with the previous observations that the catalytic AKT inhibitor capivasertib had activity against tumors having germline *PTEN* mutations and diminished/absent PTEN staining, we believe that using *PTEN* mutations was an appropriate marker of potential populations who may benefit from TAS-117 in our study.

## TAS-117 & the current trial

Herein, we describe the design and rationale for an international phase II study that is being conducted to evaluate the safety, tolerability, pharmacokinetics (PK), pharmacodynamics (PD) and antitumor activity of TAS-117 in patients with advanced solid tumors harboring germline *PTEN* inactivating mutations. As this is the first study to include patients from non-Asian populations, the initial part of the trial will reassess dosing of TAS-117 to identify an optimal dose and regimen for the global population.

## Background & rationale

TAS-117 is a non-ATP competitive, orally bioavailable, allosteric AKT inhibitor with highly potent and selective inhibition against the kinase activity of all three AKT isoforms (AKT1, 2 and 3) and no observed off-target inhibition of kinases [18]. TAS-117 also inhibits phosphorylation of downstream substrates of AKT (mTOR), proline-rich AKT substrate of 40 kDa (PRAS40), B-cell leukemia/lymphoma 2-associated death promoter (BAD), forkhead box protein O1 (FOXO1) and glycogen synthase kinase 3 [18–20].

Growth inhibition of human cancer cell lines has been shown *in vitro* following treatment with TAS-117, including breast, endometrial, lung and ovarian cancer cells [18]. In a nude mouse model, once-daily (QD) administration of TAS-117 caused significant dose-dependent, antitumor effects against NCI-N87 (gastric carcinoma) and KPL4 (breast cancer) xenografts [18].

A first-in-human phase I study in Japan (Japan Pharmaceutical Information Center [JAPIC] ID: JapicCTI-152780) investigated the safety, efficacy, PK, PD and pharmacogenomic profiles of TAS-117 in 43 patients with advanced solid tumors for whom no standard treatment remained [21]. The recommended phase II doses (RP2Ds) for Japanese subjects were identified as 16 mg QD and 24 mg/day for 4 days on/3 days off intermittent dosing, respectively. In the efficacy-evaluable patients who received 24mg 4 days on/3 days off intermittent dosing, objective responses were observed in one patient with phosphatidylinositol-4,5-bisphosphate 3-kinase catalytic subunit alpha (*PIK3CA*) mutated endometrial cancer and five patients with ovarian clear cell carcinoma. The disease control rate (DCR) was 61.5% in 13 patients with *PIK3CA*-mutated endometrial cancer, 80.0% in five patients with *AKT*-altered endometrial cancer and 37.5% in 16 patients with ovarian clear cell carcinoma. In addition, TAS-117 showed a manageable safety profile across patients with all cancer types enrolled in the study. The most common (reported in ≥30% of patients) treatment related adverse events were maculopapular rash, stomatitis, hyperglycemia and leukopenia [21].

The Korea-biomarker-driven multi-arm drug screening, knowledge and evidence-generating targeted (K-BASKET) trial (ClinicalTrials.gov ID: NCT03017521) included a phase II study of TAS-117 in patients with advanced solid tumors harboring *PI3K/AKT* gene aberrations [22]. In the study, individuals with gastrointestinal (GI) cancers were orally administered TAS-117 16 mg QD, and those with non-GI tumors were administered 24mg on a 4 days on/3 days off schedule. In total, 13 patients were enrolled: eight with non-GI (breast, ovarian, endometrial and non-small-cell lung) and five with GI (colon, rectal, gastric and gallbladder) cancers. In these patients with diverse advanced solid tumors, TAS-117 showed some antitumor activity and manageable toxicity. An objective response was observed in a patient with ovarian cancer having a *PIK3CA*^E545K^ mutation. In two patients with breast cancer harboring *PIK3CA*^H1047R^ and *AKT1*^E17K^ mutations, respectively, stable disease with tumor shrinkage was reported [22].

### Population considerations for current study

Germline mutations in *PTEN* have been reported in a variety of rare PHTS conditions and have also been found in previously undiagnosed individuals with isolated PHTS-related phenotypes (e.g., autism spectrum disorder with macrocephaly [23], breast cancer [24] and differentiated thyroid cancer [25]), suggesting that PHTS may be underdiagnosed [7]. Given that the various forms of PHTS (including CS) are very rare, we designed our study as a tumor-agnostic trial to enable potential enrollment of individuals with a variety of cancers harboring germline *PTEN* inactivating mutations. We also considered that patients harboring germline *PTEN* mutations are at increased risk of developing cancer throughout their lifetime, including cancers such as thyroid cancer, renal cancer and melanoma, which can present during childhood in patients with PHTS [11]. As the unmet medical need in pediatric patients is as high as that in adult patients with germline *PTEN* mutations, our trial aims to enroll both adolescent (aged 12–18 years) and adult patients (aged ≥18 years) with germline *PTEN* mutations. According to US FDA guidance, adolescent patients weighing ≥40 kg in oncology trials may receive the same fixed dose administered in adults [26]. Predicted TAS-117 exposures in adolescent patients weighing ≥40 kg (by allometric scaling [27]) based on PK data from the Japanese phase I study) [21] were comparable with PK results of adult patients [20].

### Study design

**Figure 2.**
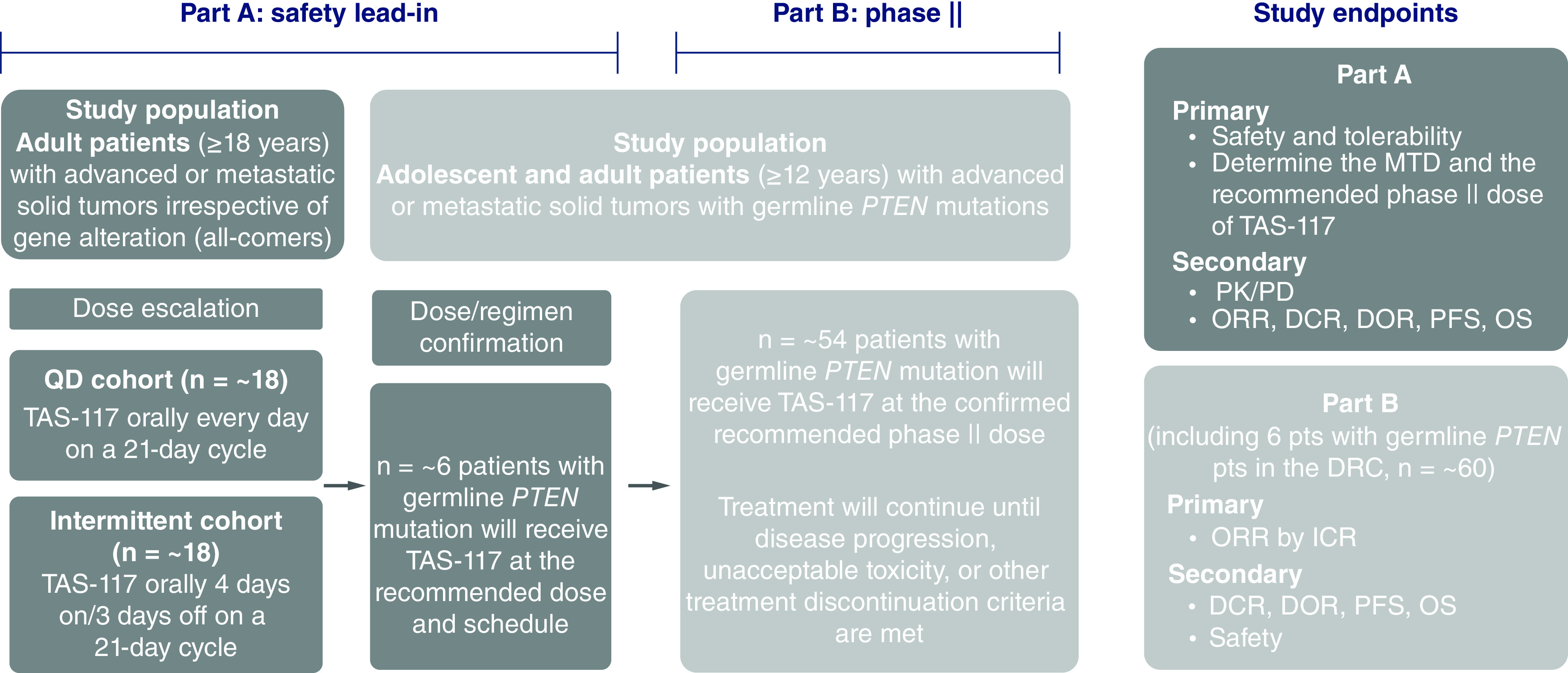
TAS-117-201 study design. DCR: Disease control rate; DOR: Duration of response; DRC: Dose and regimen confirmation; ICR: Independent central review; MTD: Maximum tolerated dose; ORR: Overall response rate; OS: Overall survival; pt: Patient; PD: Pharmacodynamics; PFS: Progression-free survival; PK: Pharmacokinetics; QD: Once daily.

TAS-117-201 is an open-label, single-arm phase II study (Figure 2) to evaluate the safety, tolerability, PK, PD and antitumor activity of TAS-117 in patients with advanced solid tumors harboring germline *PTEN* inactivating mutations. The study is being conducted in two parts: Part A: safety lead-in (dose escalation and dose/regimen confirmation); and Part B: single-arm phase II study. Dose escalation in the safety lead-in (Part A) will be carried out in a 3+3 design with QD or intermittent dosing (4 days on and 3 days off, each week), with each regimen administered on a 21 day cycle (Figure 2 & Table 2). The primary objectives of Part A are to investigate the safety and tolerability and to determine the maximum tolerated dose and the RP2D of TAS-117 (Table 3). During dose escalation, the study population will include patients with advanced or metastatic solid tumors irrespective of gene alterations. Based on data from the phase I study conducted in Japan [21], the starting dose will be 16 mg QD or 24 mg/day (intermittent), with subsequent doses increased by 4 mg, up to a maximum of 24 mg/day for QD dosing and 32 mg/day for intermittent dosing, respectively. Enrollment to the QD and intermittent dosing cohorts will occur in parallel. Dose-limiting toxicities (DLTs) will be evaluated during Cycle 1 only.

**Table 2.** TAS-117 dose-escalation levels.

Dose levels	TAS-117 dose	
	QD regimen, mg/day	Intermittent dosing (4 days on/3 days off per week), mg/day
-1	12	20
1	16	24
2	20	28
3	24	32

QD: Once daily.

After the recommended dose and regimen (either QD or intermittent dosing) are selected during dose escalation, enrollment into the dose and regimen confirmation portion of the study will commence. An additional six patients will be enrolled to further assess the safety and tolerability of the recommended dose and regimen of TAS-117 determined during dose escalation. The study population will be the same population planned for phase II of the study: patients with advanced or metastatic solid tumors (excluding primary brain tumors) harboring germline *PTEN* inactivating mutations. For part A (safety lead-in), it is planned that patients will be recruited across 12–14 countries (Austria, Brazil, France, Italy, Singapore, Japan, South Korea, Portugal, Spain, Taiwan, Sweden, UK, Denmark and USA); for part B (single-arm phase II), it is planned that trial participation will expand to include additional countries (Australia, Germany and Switzerland).

### Eligibility criteria

To be eligible for inclusion, all patients in this study must have an Eastern Cooperative Oncology Group performance status (ECOG PS) of 0 or 1 and have progressed after standard treatment for advanced or metastatic disease or have been intolerant to/ineligible for available standard therapies.

To be eligible for inclusion in part A (dose escalation, all-comers), patients must be aged ≥18 years, have histologically or cytologically confirmed advanced or metastatic solid tumors (irrespective of gene alterations) and have at least one measurable or nonmeasurable lesion per Response Evaluation Criteria in Solid Tumors version 1.1 (RECIST v1.1).

To be eligible for the dose/regimen confirmation in part A, or inclusion in part B (the single-arm phase II study), all patients must be aged ≥12 years (patients aged ≥12 and <18 years must weigh ≥40kg), have an ECOG PS of 0 or 1 (for patients aged ≥18 years) or Karnofsky performance status of ≥70% (for patients aged 12–18 years), have histologically confirmed advanced or metastatic solid tumors, have locally confirmed germline *PTEN* inactivating mutations determined from a blood sample and have at least one measurable lesion per RECIST v1.1. The availability of archived tissue blocks is essential to perform exploratory analyses planned within the study (these are outlined in the outcome measures/end points section), so patients in these later parts of the study will be required to have histological diagnosis.

Patients having any of the following criteria will be excluded from the study: history or current evidence of active interstitial lung disease or pneumonitis; current evidence of diabetes mellitus requiring insulin therapy; prior treatment with PI3K/AKT/mTOR pathway inhibitors; primary brain tumor. Patients with meningeal carcinomatosis, leptomeningeal carcinomatosis, spinal cord compression or symptomatic or unstable brain metastasis will also be excluded. Any patients with prior active malignancies will be excluded unless a complete remission was achieved prior to enrollment, and no additional therapy is required or anticipated to be required during the study.

### Planned sample size & study period

The estimated enrollment for part A (safety lead-in) is approximately 42 participants (36 patients [all-comers, irrespective of gene alteration] in the dose-escalation phase and six patients with germline *PTEN* inactivating mutations in the dose/regimen confirmation phase). The first patient was enrolled in April 2021. The estimated enrollment in part B is approximately 54 participants. It is estimated that part B will be initiated in March/April 2023. In the study overall, a total of 60 patients with germline *PTEN* inactivating mutations will be enrolled, consisting of 54 patients in part B if six patients are enrolled in the dose/regimen confirmation in part A. Once the RP2D and regimen are confirmed, part B of the study will commence following discussion between the investigators and the sponsor. It is estimated that overall study completion will occur in 2026.

### Outcome measures/end points

The primary objectives for part A are to investigate the safety and tolerability and to determine the MTD and the RP2D of TAS-117 (Table 3). Secondary objectives for part A are to characterize the PK and PD profiles of TAS-117. The primary objective of part B is to evaluate the overall response rate (ORR) in patients with solid tumors harboring germline *PTEN* inactivating mutations (including patients in the dose/regimen confirmation portion in part A) based on independent central review (ICR). The secondary objective for Part B is to evaluate the safety profile of TAS-117.

**Table 3.** Primary, secondary and exploratory objectives in TAS-117-201.

Part A	Part B (including dose and regimen confirmation in part A)
Primary objectives • To investigate the safety and tolerability and to determine the MTD and the RP2D of TAS-117	Primary objective • To evaluate the ORR in patients with solid tumors harboring germline *PTEN-*inactivating mutations based on ICR
Secondary objectives • To characterize: ○ PK profile of TAS-117 ○ PD profile of TAS-117 • To evaluate: ○ ORR (investigator assessment) ○ DCR, DOR (investigator assessments) (ICR assessment will be also performed) ○ OS	Secondary objectives • To evaluate the safety profile of TAS-117 • To evaluate: ○ ORR (investigator assessment) ○ DCR, DOR (investigator assessments) (ICR assessment will be also performed) ○ OS
Exploratory objective • To confirm *PTEN*-inactivating mutations and explore other potential predictive biomarkers of TAS-117	Exploratory objectives • To confirm *PTEN*-inactivating mutations and explore other potential predictive biomarkers of TAS-117 • To evaluate TAS-117 exposure-response relationship • To assess PROs: ○ Severity of pain and the impact of pain on daily function and assess analgesic use ○ Disease-related symptoms, impact of treatment and HRQoL based on patient-reported data ○ Current severity and overall change in health status from the start of study treatment

DCR: Disease control rate; DOR: Duration of response; HRQoL: Health-related quality of life; ICR: Independent central review; MTD: Maximum tolerated dose; ORR: Overall response rate; OS: Overall survival; PD: Pharmacodynamic; PK: Pharmacokinetic; PRO: Patient-reported outcome; PTEN: Phosphatase and tensin homolog deleted on chromosome 10; RP2D: Recommended phase II dose.

In both part A and part B, further secondary objectives will be to evaluate ORR based on investigator assessment, to evaluate DCR, duration of response (DOR) and progression-free survival based on investigator assessments (ICR assessment will also be performed in the dose/regimen confirmation portion in part A, and in part B), and to evaluate overall survival. Exploratory analyses will be performed in parts A (dose/regimen confirmation portion) and B to confirm *PTEN* inactivating mutations, and to explore other potential predictive biomarkers of TAS-117. This will include assessment of related genetic variants by next-generation sequencing, FISH, and/or immunohistochemistry and potential retrospective analysis of target engagement biomarkers, for example, AKT, pAKT, PRAS40 and/or pPRAS40. In the dose/regimen confirmation portion in part A, and in part B, additional exploratory analyses of patient-reported outcomes will be performed. Exploratory exposure–response analyses of selected efficacy and safety end points will be performed using model-estimated TAS-117 exposures.

### Study procedures

Efficacy parameters will be assessed based on on-site tumor assessments (including computed tomography or MRI) as assessed by the investigator/local radiologist and ICR according to RECIST v1.1 guidelines. Initial tumor imaging will be performed during screening (within 21 days before randomization). Thereafter, imaging will be performed every 9 weeks (±1 week) until radiologic disease progression or initiation of subsequent anticancer therapy (whichever occurs first), or withdrawal of consent. If the investigator determines that a patient has developed clinical disease progression manifested by symptomatic deterioration but not supported by radiologic evidence of progression, the patient may stop treatment.

The assessment of safety will be based on evaluation of all adverse events (AEs), including treatment-emergent AEs, treatment-related AEs, serious AEs, AEs leading to discontinuation and AEs leading to dose interruption, modification and on-study laboratory parameters. Additional safety assessments include clinical laboratory tests, vital signs and 12-lead ECGs. Grading of AEs will be performed using CTCAE version 5.0. Once a patient discontinues study treatment, during the survival follow-up, the patient or family will be contacted every 12 weeks (±2 weeks) after the last dose of TAS-117 until the study is considered complete, the patient withdraws consent or the study is terminated early by the sponsor.

### Statistics

The full analysis set will include all patients who received at least one dose of study drug (in part A or part B); these patients will be included in the safety and efficacy analysis. The DLT evaluable analysis set will include all patients in the part A dose escalation who have experienced a DLT event during the first cycle or who completed the first cycle without experiencing a DLT and with at least 80% of planned study treatments administered. The PK evaluable analysis set will include all patients who received study drug and have TAS-117 plasma concentration data. The DLT rate will be summarized descriptively at each dose level and overall in part A. ORR, DCR and DOR will be summarized descriptively.

For the analysis of ORR in patients with *PTEN* inactivating mutations in the dose/regimen confirmation portion in part A, and in part B, an ORR of ≥30% will be considered clinically meaningful. This threshold assumed that the most frequently enrolled patients are expected to be those with breast cancers, reflecting the higher prevalence of germline *PTEN* mutations observed in patients with breast cancers compared with other types of cancer [28], and the ORR for best supportive care or placebo being <10% in patients with metastatic breast cancers [29]. With a total of 60 patients (in the dose/regimen confirmation portion in part A, and in part B), if the observed ORR is 30%, the 95% CI is 18.9–43.2%.

Progression-free survival and overall survival will be estimated using the Kaplan–Meier method. In addition, the Brief Pain Inventory-Short Form will be used to assess the severity and impact of pain on daily function, and the Analgesic Quantification Algorithm score will be used to assess opiate analgesic use. Biomarker data will be summarized descriptively. The safety analysis will be performed by dose level using the full analysis set, and will involve the evaluation of AEs, laboratory values, vital signs measurements, physical exams, ECGs and concomitant medications.

## Conclusion

The serine/threonine kinase AKT is a key component of the PI3K/AKT/mTOR signaling pathway as it exerts a pivotal role in regulating cell growth, proliferation, survival and metabolism [1]. PTEN acts as a potent tumor suppressor within the PI3K/AKT/mTOR pathway by preventing buildup of phosphatidylinositol-3, 4, 5-triphosphate (PIP3), which promotes activation of AKT [5]. Loss of PTEN activity promotes AKT activation, which is associated with a shift in cellular activity toward oncogenesis [5]. Germline mutations in *PTEN* are a hallmark of PHTS, including CS [7], where they are believed to drive increased lifetime risk for cancer, and risk for developing certain cancers in childhood, such as thyroid and renal cancer [11]. In two patients with metastatic breast cancer harboring different germline *PTEN* mutations, treatment with capivasertib led to durable complete tumor responses, suggesting that AKT inhibitors provide a rational option for cancer patients harboring germline *PTEN* mutations [8]. To date, several prospective studies in patients with *PTEN* inactivation due to somatic and germline *PTEN* mutations have failed to demonstrate a clinical benefit for various agents targeting the PI3K/AKT/mTOR signaling pathway: for example, a phase II trial of the allosteric AKT inhibitor MK-2206 in patients with advanced breast cancer who had tumors with *PIK3CA/AKT1* mutations and/or *PTEN* loss/mutation found that this agent had limited clinical and PD activity as monotherapy, and the study was stopped early due to futility [30]. Similarly, a single-arm phase II trial of the mTOR inhibitor everolimus in patients with *PIK3CA* amplification/mutation and/or *PTEN* loss in advanced solid tumors refractory to standard therapy failed to meet its primary objective of demonstrating antitumor activity, and the study did not proceed to the planned second stage [31]. The TAS-117 study described here may achieve more positive findings, for example due to differences in the TAS-117 pharmacologic PK/PD profile and molecular targets versus MK-2206 and everolimus, respectively. In addition, to our knowledge, our study is the first to purely target germline *PTEN* inactivating mutations. Depending on the trial’s results, our study may support future evaluation of AKT inhibition using TAS-117 as a therapeutic strategy for solid tumors harboring somatic *PTEN* mutations.

The lack of any approved therapy to target the PI3K/AKT/mTOR pathway in pediatric and adult patients who carry germline *PTEN* mutations known to significantly increase their lifetime risk for cancer [11] remains an area of unmet medical need. In two previous clinical studies, TAS-117 has shown clinical efficacy in *PIK3CA* mutated endometrial and ovarian cancers, and in patients with ovarian clear cell carcinoma and had a manageable safety profile across patients with all cancer types enrolled in these studies [21,22].

TAS-117-201 is an ongoing, multicenter, international phase II study designed to evaluate the safety, tolerability, PK, PD and antitumor activity of TAS-117 in adolescent and adult patients with advanced or metastatic solid tumors harboring germline *PTEN* inactivating mutations. The TAS-117-201 study is being carried out in close association with the PHTS Foundation advocacy group (ptenfoundation.org), which is assisting with recruitment of patients with germline *PTEN* mutations to the study. It is hoped that our study will determine whether TAS-117 monotherapy is beneficial to patients who have advanced or metastatic solid tumors with germline *PTEN* inactivating mutations.

**Executive summary**

**Background**

- Aberrant activation of the PI3K/AKT/mTOR signaling pathway contributes to oncogenesis in solid tumors and hematologic malignancies.
- Within this pathway, phosphate and tensin homolog (PTEN) acts as a potent tumor suppressor.
- Germline mutations in *PTEN* are a hallmark of PTEN hamartoma tumor syndrome, which includes Cowden syndrome, where they appear to elevate risk of cancer during childhood and throughout life.
- There are currently no approved drugs to target the PI3K/AKT/mTOR pathway in individuals harboring germline *PTEN* inactivating mutations, so addressing these drivers of oncogenesis is an area of high unmet medical need in these adult and pediatric patients.
- In two patients with metastatic breast cancer harboring different germline *PTEN* mutations who were enrolled in clinical trials, treatment with the catalytic AKT inhibitor capivasertib led to durable complete tumor responses, suggesting that targeted AKT therapy could be an effective strategy in patients with cancer having germline *PTEN* mutations.

**TAS-117**

- TAS-117 is a novel, non-ATP competitive, orally bioavailable, allosteric AKT inhibitor with highly potent and selective inhibition against the kinase activity of all three AKT isoforms (AKT1, 2 and 3) and no off-target inhibition of kinases.
- In previous clinical studies in Asia, TAS-117 has shown clinical efficacy in *PIK3CA*-mutated endometrial and ovarian cancers, in breast cancer cases harboring *PIK3CA* or *AKT1* mutations, and in patients with ovarian clear cell carcinoma.
- We hypothesized that patients with advanced or metastatic cancer harboring germline *PTEN* inactivating mutations may benefit from monotherapy with TAS-117.

**TAS-117-201 study**

- TAS-117-201 is an open-label, single-arm phase II study to evaluate the safety, tolerability, PK, PD and antitumor activity of TAS-117 in patients with advanced solid tumors harboring germline *PTEN* inactivating mutations.
- The study has two parts: Part A: safety lead-in (dose escalation, and dose/regimen confirmation); and part B: single-arm phase II study.
- As this is the first study to include non-Asian patients, the initial part of the trial will reassess dosing of TAS-117 to identify an optimal dose and regimen for the global population.
- The unmet medical need in pediatric patients with germline *PTEN-*inactivating mutations is as high as that in adult patients, so our trial aims to enroll both adolescent (aged 12–18 years) and adult patients (aged ≥18 years) with germline *PTEN* mutations.
- The primary end point for part B is ORR based on ICR; secondary end points are safety, DCR, DOR and OS; exploratory analyses of biomarkers and PROs are also included.
- The estimated enrollment for part A is approximately 42 participants and for part B is approximately 54 participants.
